# Transcriptome Sequencing and *De Novo* Analysis of the Copepod *Calanus sinicus* Using 454 GS FLX

**DOI:** 10.1371/journal.pone.0063741

**Published:** 2013-05-06

**Authors:** Juan Ning, Minxiao Wang, Chaolun Li, Song Sun

**Affiliations:** 1 Key Laboratory of Marine Ecology and Environmental Sciences, Institute of Oceanology, Chinese Academy of Sciences, Qingdao, China; 2 Graduate University, Chinese Academy of Sciences, Beijing, China; 3 Jiaozhou Bay Marine Ecosystem Research Station, Chinese Ecosystem Research Network, Qingdao, China; Ecole Normale Supérieure de Lyon, France

## Abstract

**Background:**

Despite their species abundance and primary economic importance, genomic information about copepods is still limited. In particular, genomic resources are lacking for the copepod *Calanus sinicus*, which is a dominant species in the coastal waters of East Asia. In this study, we performed *de novo* transcriptome sequencing to produce a large number of expressed sequence tags for the copepod *C. sinicus*.

**Results:**

Copepodid larvae and adults were used as the basic material for transcriptome sequencing. Using 454 pyrosequencing, a total of 1,470,799 reads were obtained, which were assembled into 56,809 high quality expressed sequence tags. Based on their sequence similarity to known proteins, about 14,000 different genes were identified, including members of all major conserved signaling pathways. Transcripts that were putatively involved with growth, lipid metabolism, molting, and diapause were also identified among these genes. Differentially expressed genes related to several processes were found in *C. sinicus* copepodid larvae and adults. We detected 284,154 single nucleotide polymorphisms (SNPs) that provide a resource for gene function studies.

**Conclusion:**

Our data provide the most comprehensive transcriptome resource available for *C. sinicus*. This resource allowed us to identify genes associated with primary physiological processes and SNPs in coding regions, which facilitated the quantitative analysis of differential gene expression. These data should provide foundation for future genetic and genomic studies of this and related species.

## Introduction

Copepods are more abundant than any other multicellular animal group, including the hyper-abundant insects and nematodes [1], [2]. Over 12,000 validated species of copepods have been recognized, which inhabit a domain that extends from the nutrient-rich black oozes of the abyssal ocean depths to the nutrient-poor waters of the highest mountain tarns. As the dominant secondary producers of the marine ecology, copepods play important roles in aquatic food webs. They consume microorganisms and are the main food source for many commercial fishes [3], such as anchovy, cod, herring and salmon. Therefore, copepods are the linchpin of aquatic food webs and they critically support marine fish production. Copepods play important roles in the global carbon budget. Copepods transfer carbon into the deep sea via their vertical migrations between surface and deeper waters [4]. In addition, copepods are sensitive indicators of the climate because ocean warming affects the abundance, distribution and community structure of copepod [5]. Despite their species abundance, diverse geographical distribution, and global importance, limited biological information is available at the molecular level and no model species exist. To date, sequencing efforts and the application of genomic techniques have been limited to a small number of species [2], [6]. Genetic or genomic studies of a broader range of copepods would obviously facilitate future developmental, distributional, and ecological research into copepods.

Recent advances in next-generation sequencing (NGS) and bioinformatics have generated genome-level information for model and non-model organisms [7]–[9]. The increased throughput of NGS platforms, such as massively parallel 454 pyrosequencing, facilitate the rapid and cost-effective generation of massive amounts of sequence data [7], [10]. However, even with high-throughput sequencing technologies, the sequencing of complex genomes remains expensive. Transcriptome sequencing provides an attractive alternative for whole-genome sequencing because it only analyzes the transcribed portions of the genome. This strategy reduces the sequencing cost and experimental complexity, as well as improving the transcript coverage [11], [12]. In addition, this sequencing method allows the *de novo* assembly and annotation of expressed genes [13], which makes it highly suitable for non-model organisms. Indeed, transcriptome sequencing has been applied successfully to several non-model organisms, which has facilitated the detection of single nucleotide polymorphisms (SNPs), discovery and annotation of genes, analysis of differential gene expression, and functional studies of gene expression [14]–[20].

*Calanus sinicus* is a dominant copepod in the coastal waters of East Asia including China, Korea, and Japan [21]. *C. sinicus* may account for 80% of the total zooplankton abundance in the Yellow Sea [22], where it links primary production of the Yellow Sea to fish larvae and juveniles [23]. Given its ecological importance, many researchers have investigated this species in biological and ecological studies [21], [23]–[27]. However, limited genomic resources are available for *C. sinicus* so several key mechanisms remain unknown in *C. sinicus*, such as the mechanism of diapause and the population genetics of *C. sinicus*. During its life cycle, many *C. sinicus* oversummer in a diapause phase (a dormant over-summering phase where development is suppressed to adapt to the high temperature and seasonal food supply) in the Yellow Sea Cold Water Mass (YSCWM) [25], [26]. However, little is known about the triggers that initiate and terminate the diapause, or the internal processes associated with these triggers. Understanding these processes is important given that subtle changes in environmental conditions, which may affect diapause, may have consequences for the entire copepod-based ecosystem. *C. sinicus* is the dominant species in the continental shelf waters of the Northwest Pacific Ocean, such as the Yellow Sea population [26]–[28], the Bohai Sea population [28], and the Nanhai Sea population [29]. However, there is no effective molecular marker, leaving the phylogenetic relationships among populations of the species still elusive. Therefore, there is an urgent need to design more genetic tools for *C. sinicus*.

In this study, we performed *de novo* transcriptome sequencing of *C. sinicus* using the 454 GS FLX platform. The ultimate goal of this study was to produce whole-transcriptome sequences, which would provide an invaluable resource for future genetic and genomic studies of this and related species. We identified a wide diversity of candidate genes involved in all major signaling pathways and developmental processes. Given the fact that diapause occurs in copepodite stages IV and V (C4 and C5) and that these copepodites develop into adults at the end of the diapause [25], [27], we also hypothesized that key regulation genes must be involved in diapause even during the transition between active copepodites and adults. We used active copepodites (C4 and C5) and adults (males and females) as the sequencing materials, and we aimed to identify potential candidate genes that were differentially expressed between the *C. sinicus* copepodites and adults, which included genes involved in the regulation of diapause.

## Results and Discussion

### Sequencing analysis and assembly

Two types of cDNA samples, which represented different developmental stages and adult tissues of *C. sinicus*, were prepared and sequenced using the 454 GS FLX platform. The two sequencing runs produced a total of 1,470,799 reads with an average length of 355 bases (Table 1). Newbler v2.6 was used with the default parameters to screen for adapter sequences and eliminate poor quality reads. After quality trimming and removal of adapter sequences, 1,368,381 (93% of the raw reads) reads remained in the assembly. Of these, 1,123,512 (76.4%) reads assembled wholly or partially into contigs and 244,869 (16.5%) reads remained as singletons. The remaining reads were excluded because they originated from repeat regions (1,365 reads; 0.09%), were outliers (59,125 reads; 4.0%), or were too short (<40 base pairs: 41,928 reads; 2.9%). All of the raw tag data produced in this study has been deposited in the NCBI SRA database (accession number: SRA064006).

**Table 1:** Summary of the sequencing and assembly of the *C. sinicus* transcriptome.

Raw reads (bade pairs)	1,470,799(523,341,132)
Clean reads	1,368,381
Isogroup	19,149
Isotigs	31,591
Isotig N50	873
Mean # isotigs per isogroup	1.6
Contigs	56,809
Mean # contigs per isotig	2.7
Singletons	244,869

Newbler's terminology for assembled reads comprised three elements: contigs (stretches of assembled reads that were free of branching conflicts), isotigs (continuous path through a set of contigs), and isogroups (groups of isotigs arising from the same set of contigs). For consistency, we use this terminology throughout this paper. Our data were assembled into 56,809 contigs, which grouped into 31,591 isotigs. Of these isotigs, 14,791 (46.8%) contained only one contig, and the average number of contigs per isotig was 2.7. The isotig N50 length was 873 bp, which means that 50% of the bases were incorporated into isotigs ≥873 bp. The 31,591 isotigs fell into 19,149 isogroups, where 14,819 (77.4%) contained only one isotig (the average number of isotigs per isogoup was 1.6, Table 1).

**Figure 1:**
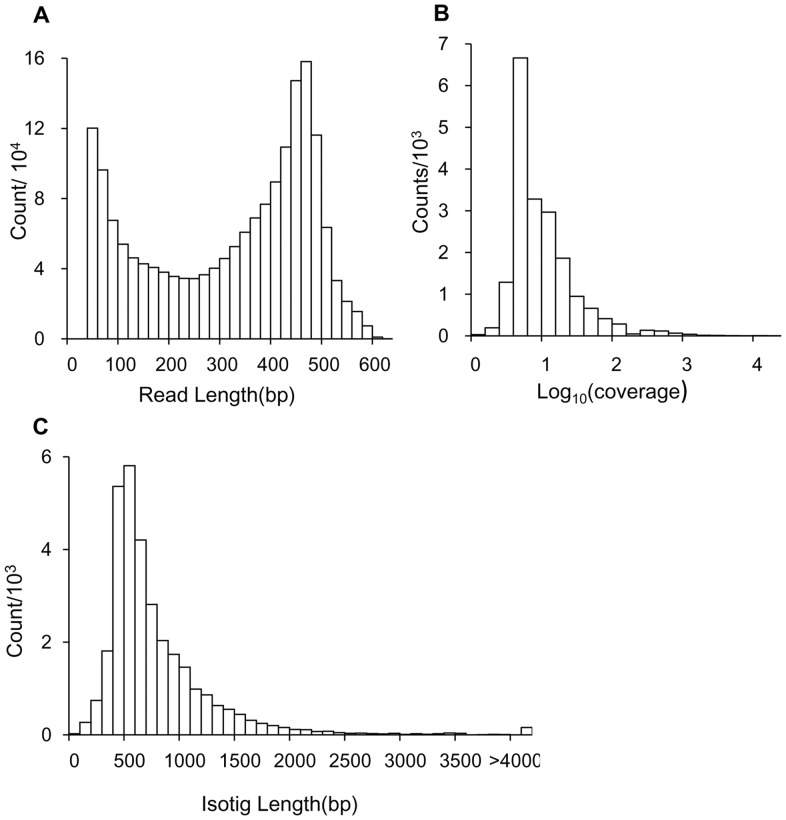
Overview of *C.* *sinicus* transcriptome sequencing and assembly. (A) Size distribution of 454 sequencing after removal of adapter and short reads (<40 bases). (B) Sequence coverage. (C) Size distribution of isotigs.

The average coverage among contigs was 32.1 reads/bp (median coverage  = 7.0 reads/bp, Figure 1B), which means that every base pair in the transcriptome was sequenced 32.1 times on average. This coverage value was much higher than that in previous studies [16], [30], [31] and it should be helpful for distinguishing SNPs from potential sequencing errors in raw reads [32].

### Transcriptome annotation

Several complementary approaches were used to annotate the assembled sequences. First, we used BLASTX to map the 276,460 assembled sequences (31,591 isotigs + 244,869 singletons) against the entire RefSeq protein database with an E-value cut-off of 1e -3. To simplify the statistics, we only reported the results for the longest isotig per isogroup. Of the 19,149 isotigs, 9,497(49.5%) had at least one hit and 8,340 (43.5%) were matched to proteins with known functions. Of the 244,869 singletons, 71,562 (29.2%) had hits and 62,451 (25.6%) were matched to known proteins. These values were higher than those in the comparable BLAST results from most other published studies using 454-generated *de novo* transcriptomes [16], [20], [31]. This may be attributed to the deeper sequencing, which increased the length of the assembled sequences and accordingly made the sequences more likely to be identified using BLAST. The unidentifiable sequences may originate from untranslated regions (UTRs) or non-conserved portions of protein-coding sequences.

**Figure 2:**
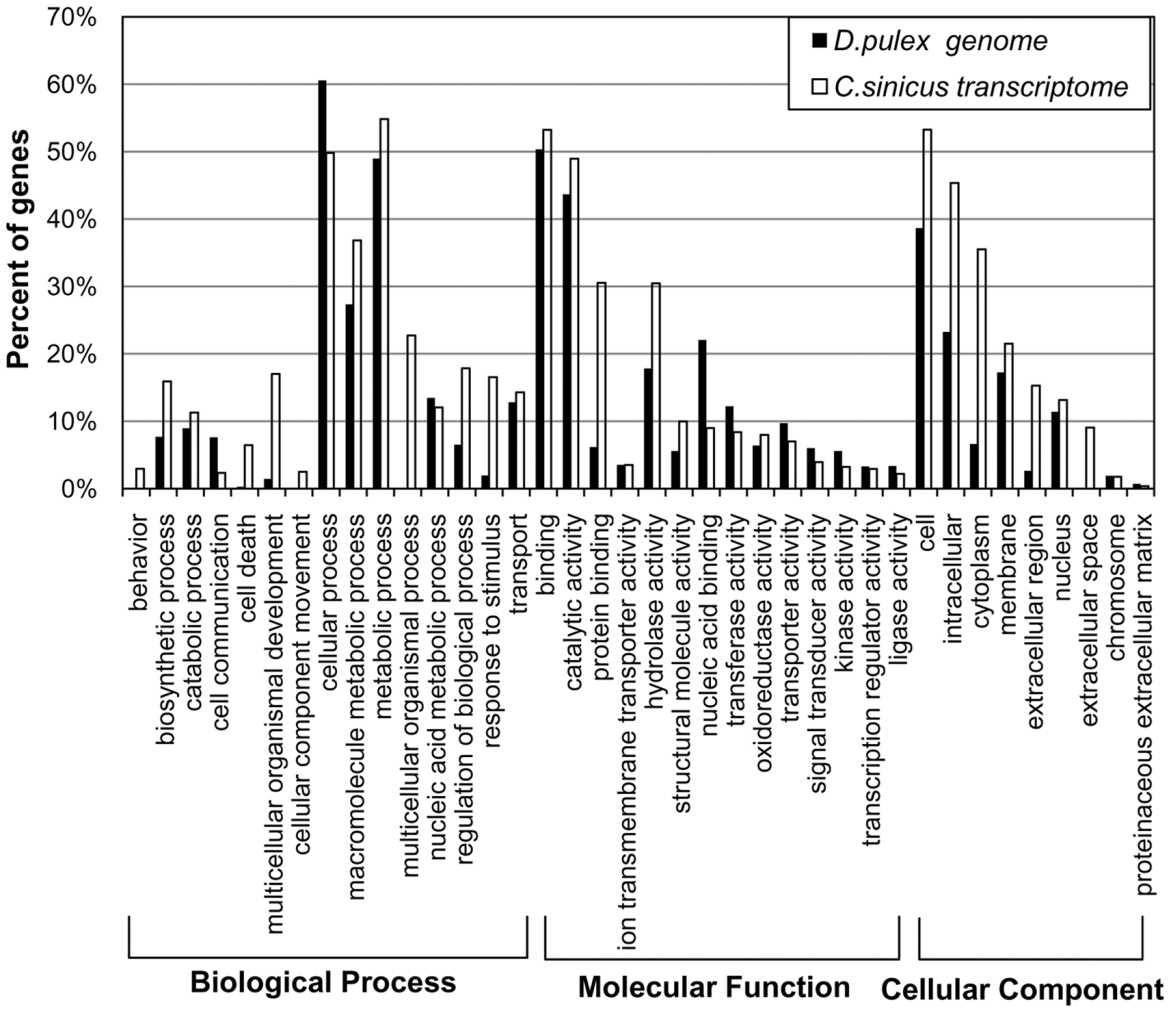
GO term distribution of BLAST hits from the *C.* *sinicus* transcriptome. Selected GO categories are shown within the top-level divisions of Biological Process, Molecular Function, and Cellular Component. The relative percentages of genes falling into GO categories are comparable between our *C. sinicus* transcriptome (white) and the *D. pulex* genome (black).

Gene Ontology (GO) analysis was carried out to explore and summarize the functional categories of the genes sequenced in this study. Of the annotated sequences, 25,676 were assigned GO terms. In total, 175,835 GO terms were obtained, which included 41.3% related to biological processes, 32.0% to molecular functions, and 26.7% to cellular components (Figure 2). The percentages of annotated *C. sinicus* sequences assigned to GO terms were compared with those from *Daphnia pulex*, the only crustacean with a sequenced genome. We found no significant differences in the percentages of genes with examined GO term categories between the *C. sinicus* transcriptome and the *D. pulex* genome, which suggests that there was a similar distribution of genes in the different functional categories, while the sequenced *C. sinicus* transcriptome did not lack major functional categories of genes.

Final functional classification and pathway assignment were performed using the Kyoto Encyclopedia of Genes and Genomes (KEGG) database. The annotated sequences were compared using bi-directional BLAST with an E-value of 1e -3 against the KEGG database. Of these sequences, 8,962 had significant matches in the database. Among the matched sequences, 2,866 sequences with enzyme commission (EC) numbers were assigned to metabolic pathways (Table 2). The metabolic pathways that were well represented in the *C. sinicus* sequences were carbohydrate metabolism, energy metabolism, amino acid metabolism, and lipid metabolism. Given the important roles of lipids in the lifecycle of copepod [33], especially during diapause, we focused greater attention on lipid metabolism. Genes were found in several pathways involved with fatty acid biosynthesis, such as fatty acid elongation, steroid biosynthesis and ether lipid metabolism (Table S1).

**Table 2:** KEGG biochemical mappings for *C. sinicus*.

KEGG pathway	Number of genes	Number of sequences (Isogroup + singleton)
**Metabolism**	**1034**	**2866**
Carbohydrate Metabolism	182	570
Energy Metabolism	142	435
Lipid Metabolism	131	324
Nucleotide Metabolism	82	302
Amino Acid Metabolism	154	389
Metabolism of Other Amino Acids	58	135
Glycan Biosynthesis and Metabolism	95	201
Metabolism of Cofactors and Vitamins	80	163
Metabolism of Terpenoids and Polyketides	28	56
Biosynthesis of Other Secondary Metabolites	27	81
Xenobiotics Biodegradation and Metabolism	55	210
**Genetic Information Processing**	**527**	**994**
Transcription	93	142
Translation	199	409
Folding, Sorting and Degradation	183	299
Replication and Repair	52	144
**Environmental Information Processing**	**264**	**612**
Membrane Transport	24	39
Signal Transduction	178	482
Signaling Molecules and Interaction	62	91
**Cellular processes**	**372**	**791**
Transport and Catabolism	167	348
Cell Motility	47	84
Cell Growth and Death	81	211
Cell Communication	77	148
**Organismal Systems**	**581**	**1499**
Immune System	102	339
Endocrine System	99	254
Circulatory System	41	91
Digestive System	92	234
Excretory System	50	81
Nervous System	114	346
Sensory System	15	31
Development	51	94
Environmental Adaptation	17	29
**Total**	**2778**	**6762**

### Estimating the number of genes expressed in *C. sinicus*

One of the primary goals of transcriptome sequencing projects is to determine the number of expressed genes. The exact gene number is indeterminable if there is no fully sequenced genome. However, several alternative strategies are available to estimate the number of genes expressed in non-model organisms. First, gene numbers can be estimated based on genes that are well matched by sequences. Thus, gene names were assigned to assembled sequences based on the gene product and gene name annotation of the best BLAST match for that sequence. This procedure successfully assigned gene names to 70,791 sequences in the total dataset, i.e., 65,147 of the sequences were ≥300 bp in length and 1,862 of the sequences were ≥1000 bp in length. Among the 70,791 annotated best hits, 14,279 different gene names were assigned. This provided a rough estimate of the number of different genes expressed in the *C. sinicus* transcriptome. Given that many sequences lacked matches in public sequence databases and thus were not assigned gene names, this number was probably an underestimate. The gene number can also be estimated based on the number of isogroups. Isogroups are groups of isotigs that arise from the same set of contigs, where each isotig in the isogroup represents a transcript variant. Therefore, isogroups may represent putative genes. We identified 19,149 isogroups in our transcriptome, which provided another estimate of the gene number in the *C. sinicus* transcriptome. No specific solutions are available for determining the number of genes without a sequenced genome, but the estimates described above suggest that over 15,000 different genes were expressed in *C. sinicus*. This estimate was close to that reported in a previous transcriptome study of another marine copepod, *Tigriopus californicus* [19], which identified 15,402 unique unigenes in the *T. californicus* transcriptome.

### Genes involved with development

The growth and development of many copepods such as *C. sinicus,* which is an important link in the food chain between phytoplankton and planktivorous fish, are of particular interest to researchers. In this sequencing project, we identified a wide diversity of candidate genes involved with developmental processes based on BLAST, GO, and KEGG annotations. Among these genes, we identified different groups of growth factors and their receptors involved with cell growth (Table 3).

**Table 3:** Selected development process genes identified in the *C. sinicus* transcriptome.

Process	NumIsotig	Length(range)
**Growth**		
ATP-binding cassette	7	415–1579
prosaposin	1	465
saposin	10	388–1856
vitellogenin receptor	2	488–2772
histone-lysine N-methyltransferase	7	588–1583
serine/threonine-protein kinase PAK 2	16	357–2002
pre-mRNA-processing factor	3	743–1029
calsyntenin	1	1206
60S ribosomal protein L12	1	494
DNA methyltransferase 1-associated protein 1	1	602
chromodomain-helicase-DNA-binding protein	1	895
transcription factor family member	92	403–1285
insulin receptor	1	729
DNA-binding protein A	1	895
elongation factor	33	356–1885
actin	196	331–1342
ubiquitin-conjugating enzyme E2 E3	14	378–1810
adenosylhomocysteinase	6	200–1665
menin	2	1084–2603
histone acetyltransferase	3	663–1486
**Molting**		
CYP315A1; ecdysteroid 2-hydroxylase	1	1390
CYP306A1	3	485–507
CYP302A1	5	406–490
CYP305A1	2	138–151
ecdysone-induced protein 75(E75)	1	747
ecdysone receptor	4	305–2056
ecdysoneless	1	2056
CYP15A1	10	390–1610
farnesoic acid O-methyltransferase(FAMeT)	6	451–1175
juvenile hormone-inducible protein	2	492–1349
juvenile hormone esterase	6	652–1924
juvenile hormone epoxide hydrolase	8	414–513
ferritin	56	247–773
elongation of very long chain fatty acids protein(ELOV)	10	365–1208
fatty acid binding protein(FABP)	17	467–747
short-chain dehydrogenase/reductase (SDR)	6	449–961

Molting and diapause are key biological processes in the life cycle of many insects and copepods. In arthropods, ecdysteroids and juvenile hormone (JH) are key molecules that regulated growth (molting), sexual maturation and egg production [34], [35]. To identify genes associated with molting and diapause, the *C. sinicus* transcriptome was searched for key ecdysteroid- and JH-related genes. We detected the expression of many known ecdysteroid biosynthesis genes, including members of the cytochrome P450 family, such as CYP315A1, CYP306A1, CYP302A1, and CYP305A1 (Table 3). We also identified transcripts for ecdysone-regulated genes, including E75, ecdysone receptor and ecdysoneless, which are known to play key roles during molting [36]–[38]. JH biosynthesis and response genes were also identified. JH is a key hormone in the regulation of the insect's life history, which maintains the larval state during molts and that directs reproductive maturation [39]. Farnesoic acid O-methyltransferase (FAMeT) and CYP15A1 play significant roles in catalysis during JH biosynthesis pathways [40], [41]. Ferritin and several other genes (ELOV, FABP, SDR) involved in fatty acids metabolism were also identified. These genes were differentially expressed in diapausing and active *Calanus finmarchicus* [42].

### Differentially expressed genes

Of the whole transcriptome sequences, 959,123 reads were generated from the *C. sinicus* copepodid larval samples and 842,238 reads were from the adults. We found that 3,798 gene sequences were differentially expressed between the two samples i.e., 1,841 sequences were categorized as up-regulated genes (those with higher expression levels in copepodite samples compared with adult samples), and 1,954 sequences were categorized as down-regulated genes (those with significantly higher expression levels in adults compared with copepodite). Of the 3,798 differentially expressed sequences, we predicted annotations for 1,503 sequences and matched about 640 different proteins (Table S2). GO enrichment of these differentially expressed sequences detected several overrepresented GO categories (Table S3), such as transport process, binding, and motor activities.

With the ultimate goal of fully understanding the molecular mechanism of diapause in *C. sinicus*, we tried to identify genes involved with the regulation of diapause. Several genes, such as JH, ecdysone, heat shock protein (HSP), ferritin, cytochrome P450, are known to play important roles in diapause regulation [42]–[44]. From all of the genes that were differentially expressed in *C. sinicus* copepodites and adults, we found several genes that may be involved with diapause (Table 4). Elongation of very long chain fatty acids protein (ELOV) and cuticle protein (CP) were up-regulated in copepodites. ELOV facilitates the regulatory step during fatty acid elongation in mammals [45]. The specific role of ELOV is unknown in *C. sinicus* but it was reported that ELOV was involved with the biosynthesis of JH [46] and the synthesis of storage lipids [42]. High expression of ELOV suggested that more fatty acids were synthesized in copepodid larvae (copepod stages IV and V), some of which were stored and prepared for later diapause. This hypothesis was consistent with the findings of a previous study where the population of *C. sinicus* on the continental shelf of the Yellow Sea during May to June exhibited features of preparation for diapause in the Yellow Sea Cold Water Mass, such as the accumulation of lipids in the oil sac and the dominance of C5s [47]. Cuticle protein is one of the structural proteins that comprises the stratum corneum together with chitin [48]. Cuticle protein was found to be highly expressed prior to the final molt and prior to the post-molting growth of adult female salmon louse (*Lepeophtheirus salmonis*) [49].

**Table 4:** Expression levels of genes that may be invovled with the diapause regulation of *C. sinicus*.

Gene	Gene length/bp	RPKM		p-value	Regulation
		Copepodite	Adult		
Cuticle protein	1568	42	0	8.10E–11	up
Ferritin	754	70	505	7.35E–38	down
Heat shock protein	1003	15	63	1.54E–05	down
ELOV	1006	301	6	1.38E–44	up
Cytochrome P450	1763	25	7	0.00399	up/down*
Juvenile hormone esterase	1924	0	31	6.64E–10	up/down
FAMeT	1175	47	77	0.02038	up/down
Ecdysteroid receptor	2056	13	2	0.00343	up/down
Retinoid X receptor	747	25	27	0.85347	up/down

* up/down: Both up and down regulated sequences were found in *C. sinicus* copepodite or adults. ELOV: elongation of very long chain fatty acids protein. FAMeT: farnesoic acid O-methyltransferase. Gene expression level is calculated by using reads per kilobase of the transcript per million mapped reads (RPKM).

The expression levels of ferritin and heat shock protein were high in adults. As an iron storage protein, ferritin plays a key role in iron metabolism [50] and protects proteins against oxidative damage [51]. In *C. finmarchicus*, ferritin was up-regulated in diapausing copepodites, where it was predicted to protect proteins and help to delay development [42]. Heat shock proteins (HSPs) are a superfamily of molecular chaperones that regulate diapause in some insects and copepods [52]. HSPs prevent irreversible protein denaturation during stress, facilitate protein refolding or destruction, and are required for stress tolerance [53]. HSP22 was found to be highly expressed in diapausing *C. finmarchicus* and was predicted to protect proteins from degradation during diapause [44].

Other proteins, such as cytochrome P450, FAMeT, ecdysteroid receptor, and retinoid X receptor were also found to have key roles in diapause regulation in other species [42], [52]. However, these proteins were not differentially expressed in the active copepodites and adults of *C. sinicus*. We will pay close attention to these genes in future expression profile analyses of active and diapause samples, the transcriptomes of which have been sequenced using the Solexa platform.

### Real-time PCR

**Figure 3:**
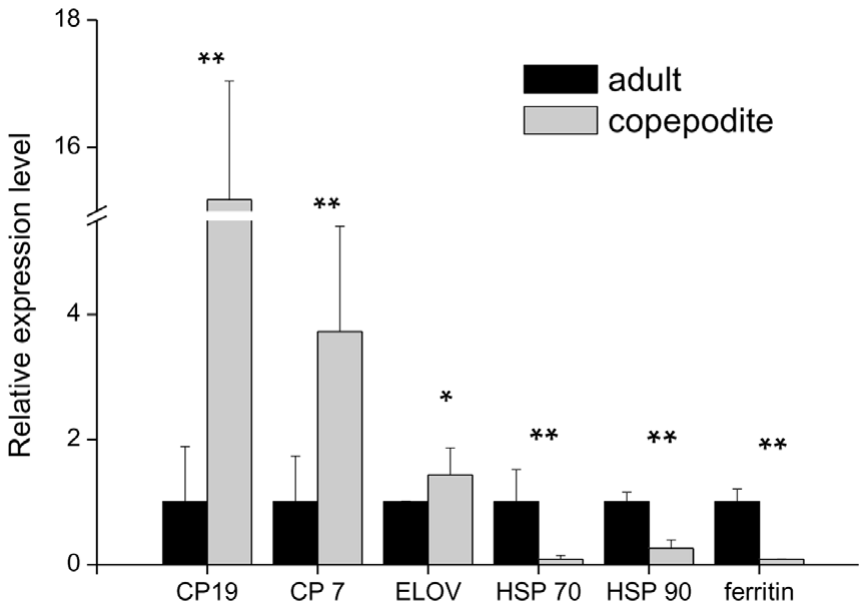
Real-time PCR validation of differentially expressed genes that may be involved in diapause. The values were normalized against actin. Significant levels were *P<0.05, **P<0.01, and values indicate the mean ± S.E.

The significant up- and down-regulation of expressed genes involved with diapause were validated by real-time PCR. The materials used in this experiment were the same as those used in 454 sequencing. The sequences of the primers used are listed in Table 5. The expression levels of the six genes validated using real-time PCR confirmed the robustness of the 454 sequencing results (Figure 3).

**Table 5:** Oligonucleotide primer sequences for real time PCR.

Gene	Primer sequence	Product size(bp)
ELOV	F: 5′ ACGGTTCTCATCTATTGCT 3′	149
	R: 5′ ATCGCAATCAATTTCGGGAC 3′	
Actin	F: 5′ GAAGATCTGGCATCACACC 3′	109
	R: 5′ CATCTTCTCTCTGTTTGCCTT 3′	
Cuticle protein 7	F: 5′ GAGGATCCAGCACACCAA 3′	118
	R: 5′ AACCGCATTTCCATATCCAC 3′	
Cuticle protein 19	F: 5′ CCCTCAGCCATTTGCCTA 3′	63
	R: 5′ TTCTTGAAGTTAGCCTTAGAGT 3′	
Ferritin	F: 5′ AACCGTGATGATCAAGCTC 3′	104
	R: 5′ CGCTTGGTCTGATATTCCAT 3′	
HSP 70	F: 5′ ATCTTCGAGGTCAAGTCCAC 3′	116
	R: 5′ TTCATGTCCTTCTTGTGCTT 3′	
HSP 90	F: 5′ TGGTTTCTACTCCGCCTA 3′	78
	R: 5′ CAGACATATTGCTCGTCA 3′	

### SNP discovery

**Figure 4:**
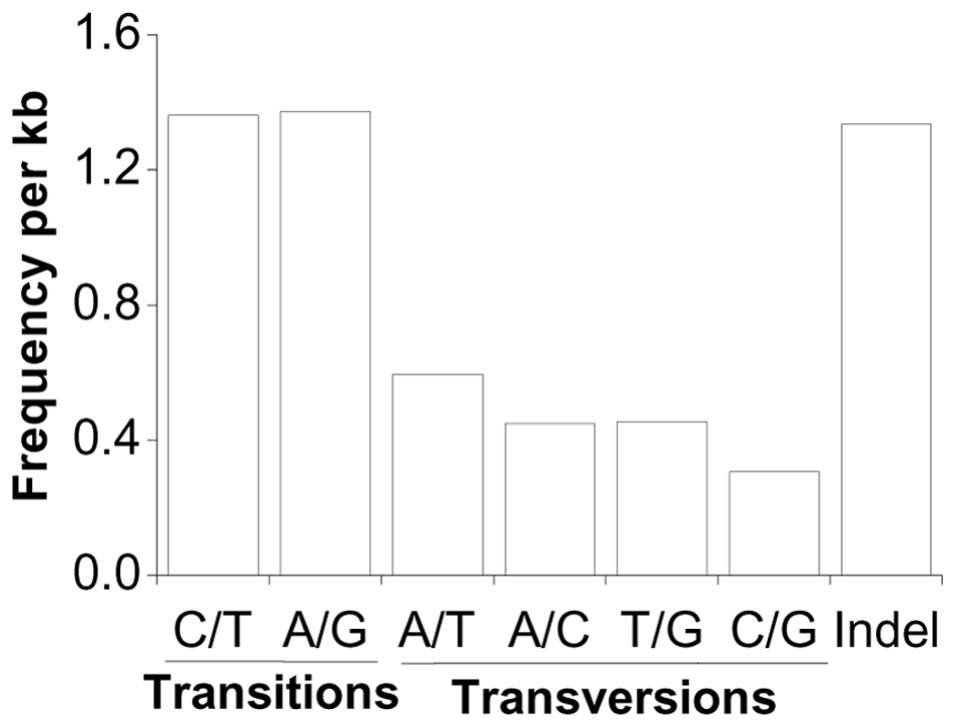
Classification of single nucleotide polymorphisms (SNPs) indentified in the ***C.*** *sinicus* transcriptome. The overall frequency of these SNPs is one per 179 bp.

Potential SNPs were detected using CLC Genomics Workbench software. We identified 284,154 high-quality SNPs and 30,409 indels from 18,891 isotigs (Figure 4). The predicted SNPs included 179,345 transitions and 132,756 transvertions. The overall frequency of all SNP types in the transcriptome, including indels, was 1 per 179 bp. These potential SNPs provide an extensive set of genetic markers for copepods and will facilitate future studies of genetic connectivity and genetic mapping at a previously unprecedented level of detail.

*C. sinicus* dominates the continental shelf waters in the Northwest Pacific Ocean. The population structure of *C. sinicus* is central to understanding the mechanism of its population recruitment. However, there are no effective molecular markers, which make it difficult to determine the phylogenetic relationships among the populations of this species. Wang reported that, compared with other gene markers (COX1 and ITS), more information could be obtained using SNP gene markers in population studies of *C. sinicus* [54]. Using the abundance of SNPs determined by 454 sequencing, we will select effective SNPs for studying the population structure of *C. sinicus*.

## Conclusions

In this work, we performed *de novo* transcriptome sequencing of *C. sinicus* using the 454 GS FLX platform. We identified many candidate genes that were potentially involved with growth, molting, and lipid metabolism. This set of candidate genes is valuable for further research. We produced a large set of Expressed Sequence Tags (ESTs) and SNPs and made them publicly available for marker development. Our comparative sequencing of copepodid larvae and adults has provided information about the gene variations involved with a number of pathways, which might potentially reflect developmental changes.

## Materials and Methods

### Sample preparation and sequencing

Copepods were collected from Jiaozhou Bay, China, and brought to the laboratory in fresh seawater. The studies were performed according to the Institute of Oceanology, Chinese Academy of Sciences guidelines for the use and care of laboratory animals in research. All necessary permits were obtained for the field studies. Copepods were collected by a 500 µm mesh zooplankton net (mouth opening 0.5 m^2^) and preserved in fresh seawater temporarily. All tows were carried out vertically from 4 m above the bottom to the sea surface. Adults (females and males) and copepodid larvae (C4 and C5) were collected immediately and stored separately in 4 L of 0.45 μm-filtered seawater at 15°C for 24 hours. Using an anatomical lens copepods were picked up at different developmental stages with adults of different sexes, including copepod stages C4 and C5, and female, and male adults. Copepods were put in 1.5 ml frozen tubes and then seawater was removed using bibulous paper. All samples were flash frozen and stored in liquid nitrogen until analysis.

Total RNA was extracted from each of the four samples (>50 mg wet weight each) using a RNeasy Mini Kit (Qiagen, Valencia, CA, USA) and purified further with a TURBO DNA-free Kit (Ambion, Austin, TX, USA). The quantity and quality of total RNA was analyzed using a 1% agarose gel and an Agilent 2100 bioanalyzer. The RNA from C4 and C5 were pooled in equal proportions to produce the copepodite sample while the adult males and females were pooled equally to produce the adult sample. Equal total RNA were reverse-transcribed into cDNA using a SMART PCR cDNA Synthesis Kit (Clontech Laboratories, Inc. CA, USA). Sample of copepodid stages (C4 and C5) was run on a sequencing plate and adult sample was run on another sequencing plate. The 454 sequencing experiments were performed by the Chinese National Human Genome Center.

### Sequence data analysis and assembly

Prior to assembly, adapter sequences and low-quality sequences were trimmed from the raw reads. All reads shorter than 40 bp were removed based on an assumption that small reads would fail to assemble and that they might represent sequencing artifacts. The trimmed and size-selected reads were assembled using the Newbler assembly program (v2.6) where all of the parameters were set to their default values. Singletons, i.e., reads that were not incorporated into any contigs, were retained in the data set because many were likely to be fragments of low-expressed transcripts. Assembled contigs and singletons were pooled for subsequent analyses.

### Sequence annotation

Before the gene name annotation, the assembled sequences were mapped against Swiss-Prot and the NCBI non-redundant (Nr) protein databases using BLASTX with an E-value threshold of 10^−3^. Gene names were assigned to each assembled sequence based on the best BLAST hit.

GoPipe [55] was used to annotate the assembled sequences with Gene Ontology (GO) terms describing biological processes, molecular functions, and cellular components. GoPipe is a tool for integrating BLAST results into streamlined GO annotations for batched sequences. We used a precomputed GO annotation from the *D. pulex* genome [56] to compare sequences with different GO annotations in *C. sinicus* and *D. pulex*.

Finally, the assembled sequences were compared to the KEGG database [57]. KEGG pathways were assigned to the assembled sequences using the online KEGG Automatic Annotation Server (KAAS), http://www.genome.jp/kegg/kaas/. The bi-directional best hit (BBH) method was used (with an E-value of 1e -3) to obtain KEGG Orthology (KO) assignment. Using the KEGG database, we further studied the complex biological behaviors of genes and determined pathway annotations for sequences.

### Differential gene expression analysis

The expression level of a transcript was quantified in reads per kilobase of the transcript per million mapped reads (RPKM) in the transcriptome [58]. Differentially expressed genes were identified in *C. sinicus* copepodid larvae (C4 and C5) and adults (male and female) by a DEGseq package using the MARS (MA-plot-based method with Random Sampling model) method [59]. Genes were regarded as differentially expressed if they exhibited two fold or greater change using a 0.1% or less false discovery rate (FDR). Differentially expressed genes were regarded as up-regulated if the expression levels in copepodid larval samples were significantly higher than those in adult samples. Down-regulated genes were those with significantly higher levels of expression in adults compared with larvae.

A Fisher's exact test was used with a threshold of 5% as the false discovery rate to determine the differentially regulated genes in the two samples for each GO term.

### Real-time PCR

The significant up- or down- regulated expressed genes associated with diapause were validated and quantified by real-time PCR. Actin, which has been used for the real-time PCR of copepod genes by other workers [42], was chosen to normalize the target gene quantities. Oligonucleotide primers were designed using the 454 sequencing data to target 75–150 bp amplicons (Table 5). The total RNA of copepodite samples and adult samples were prepared and analyzed as RNA used in 454 sequencing. The same amount of total RNA from each sample was reverse transcribed into cDNA using a RevertAid Frist Strand cDNA synthesis kit (Fermentas, Ontario, Canada).

Real-time PCR was performed using the SYBR Green® real-time PCR assay with an Eppendorf Mastercycler® ep realplex S. All samples were run in duplicate wells for each gene on a single plate. The PCR mixture contained 10.0 μL of SYBR Premix Ex Taq (Takara, Dalian, China), 0.4 μL (each) of forward and reverse primer (10 mM), 2 μL of 1∶10 diluted cDNA, and 7.2 μL of RNase-free water. The PCR conditions were as follows: 95°C for 30 s; 40 cycles of 95°C for 5 s and 60°C for 20 s. After 40 cycles, the PCR products from each reaction were subjected to melt-curve analysis to ensure that only a single product was amplified. To confirm that the correct amplification had occurred, the PCR products were analyzed by electrophoresis and PCR product sequencing. To check the amplification efficiencies, four different dilutions (of 5, 5∧2, 5∧3 and 5∧4) were tested using cDNA for the target and internal control genes.

The statistical significance of the data were tested using one-way ANOVA and independent sample t-tests. Significance was accepted at ≤0.05. The statistical analyses were performed using SPSS 18.

### SNP discovery

CLC Genomics Workbench software was used to detect potential SNPs in isotigs with sufficient depth of coverage. SNPs were identified using an arbitrary criterion of at least 4 reads supporting the consensus or variant and a similarity of 95%.

## Supporting Information

Table S1 **KEGG pathway annotation of genes involed in the lipid metabolism.**
(XLSX)

Table S2 **Genes differentially expressed in** ***C. sinicus*** **copepodid larvae and adults.**
(XLSX)

Table S3 **Go terms of genes differentially expressed in** ***C. sinicus*** **copepodites and adults.**
(DOC)
