# Supplementary material for: Transcriptome Sequencing and De Novo Analysis of the Copepod Calanus sinicus Using 454 GS FLX
Source: PLoS One. 2013 May 6;8(5):e63741. doi: 10.1371/journal.pone.0063741 (PMC3646036; doi:10.1371/journal.pone.0063741)
Supplement: Table S3 — Go terms of genes differentially expressed in C. sinicus copepodites and adults. (DOC) [file pone.0063741.s003.doc]

**Table 4.** GO terms of genes differentially expressed in *C. sinicus* copepodid larvae and adults.

| **GO Term** | **GO ID** | **FDR** |
| --- | --- | --- |
| **Biological process** |  |  |
| Transport | GO:0006810 | 0.003352 |
| **Molecular function** |  |  |
| Binding | GO:0005488 | 8.28E-25 |
| Motor activity | GO:0003774 | 5.69E-10 |
| Structural molecule activity | GO:0005198 | 0.001862 |
| Transporter activity | GO:0005215 | 0.00583 |
| **Cellar component** |  |  |
| Cytoplasm | GO:0005737 | 0.000134 |
| Intracellular | GO:0005622 | 0.000884 |
